# Coffee or Tea? A prospective cohort study on the associations of coffee and tea intake with overall and cause-specific mortality in men versus women

**DOI:** 10.1007/s10654-018-0359-y

**Published:** 2018-01-27

**Authors:** Piet A. van den Brandt

**Affiliations:** 1 Department of Epidemiology, GROW-School for Oncology and Developmental Biology, Maastricht University Medical Centre, PO Box 616, 6200 MD Maastricht, The Netherlands; 2 Department of Epidemiology, CAPHRI-School for Public Health and Primary Care, Maastricht University Medical Centre, PO Box 616, 6200 MD Maastricht, The Netherlands

**Keywords:** Coffee, Tea, Mortality, Neoplasms, Cardiovascular diseases, Cohort studies

## Abstract

Coffee and tea intake have been associated with reduced mortality, but no studies have investigated possible substitution effects. The relationship of mortality with coffee, tea, and substituting coffee with tea was investigated in the Netherlands Cohort Study. In 1986, 120,852 men and women aged 55–69 years provided information on dietary and lifestyle habits. Mortality follow-up until 1996 consisted of linkage to Statistics Netherlands. Multivariate case-cohort analyses were based on 8665 deaths and 3166 subcohort members with complete data on coffee, tea and confounders. Higher coffee intake was significantly, nonlinearly related to lower overall and cause-specific mortality in women. In men, coffee was significantly positively related to cancer and cardiovascular mortality, and inversely to respiratory and other causes of death. Tea intake was significantly, nonlinearly related to lower overall, cancer and cardiovascular mortality in men, but showed no association with mortality in women. In substitution analyses, increasing the proportion tea (replacing coffee with tea) was significantly and nonlinearly related to lower overall, cancer and cardiovascular mortality in men, but in women higher tea proportions were positively associated with overall mortality (and most causes of death). This study suggests that for men, compared to exclusive coffee drinkers, those drinking 30–50% tea showed the lowest mortality; any tea drinking seemed better than only coffee. For women, those who drank exclusively coffee or drinking up to 40% tea had the lowest mortality, but those drinking higher percentages of tea were at increased mortality risk [HR = 1.41 (95% CI 1.01–1.99) for 80–100% tea compared to exclusive coffee drinkers].

**Electronic supplementary material:**

The online version of this article (10.1007/s10654-018-0359-y) contains supplementary material, which is available to authorized users.

## Introduction

Coffee and tea are among the most widely consumed beverages in adults worldwide [1, 2]. Coffee consumption is very popular and still increasing, with the highest per capita consumption in Scandinavian countries in 2013 [3]. Tea (black or green) is worldwide the most commonly consumed beverage after water, with high per capita consumption observed in Turkey, Iran, and United Kingdom [3]. There is a long-standing interest in the health effects of coffee and tea. Epidemiological studies have been conducted on coffee or tea intake and disease incidence (e.g. cancer, CVD, diabetes) and mortality. A recent meta-analysis on coffee and mortality in 31 cohort studies [4] showed decreased overall and cardiovascular disease (CVD) mortality for coffee consumption of up to 4 cups/day. Two recent large cohort studies also showed that coffee consumption was associated with reduced risk of death (overall and from various causes) in the European Prospective Investigation into Cancer (EPIC) [5] and among African Americans, Japanese Americans, Latinos and whites in the Multiethnic Cohort (MEC) [6]. For tea, a recent meta-analysis using 18 cohort studies found that black tea consumption was significantly inversely associated with overall and cancer mortality, while green tea consumption was significantly inversely associated with overall and CVD mortality [7]. Only in a limited number of the previous cohort studies on mortality, analyses for coffee and tea intake were mutually adjusted.

While coffee and tea are both drunk in most countries, usually one predominates because of economic (e.g., trade and income), marketing, cultural and ethnic reasons [8]. Many people can choose between coffee and tea, and drink these in varying ratios depending on taste preference, lifestyle, socio-economic factors, genetics and health, but in very few mortality studies associations with coffee and tea have been investigated simultaneously, and no studies have been conducted on possible effects associated with substituting one beverage for the other. This study first evaluated the mutually-adjusted associations between coffee and tea intake with overall and cause-specific mortality in men and women, and then focused on substituting one beverage for the other, as well as on the combined effects of coffee and tea.

## Methods

### Study design and mortality follow-up

The NLCS started in September 1986 and includes 58,279 men and 62,573 women aged 55–69 years [9]. At baseline (September 1986), they completed a mailed, self-administered 11-page questionnaire on cancer risk factors. The NLCS study was approved by the Maastricht University institutional review board. For efficiency, we applied the nested case-cohort method [10], requiring only data-entry of questionnaires (which could not be scanned) of cases and a random subcohort. Following this method [9], cases were enumerated from the entire NLCS-cohort of 120,852 (numerator information of mortality rates), whereas the accumulated person-years at risk in the cohort were estimated using a subcohort of 5000 subjects (denominator information). The case-cohort method implies that the persontime at risk is estimated through a sample of the the total cohort, instead of actively following the total cohort. Data entry of questionnaires is only needed for cases and subcohort members, instead of the total cohort [9, 10].Immediately after the NLCS-baseline measurement, the subcohort (2411 men, 2589 women) was randomly sampled from the cohort, and actively followed up since 1986 for vital status and migration. For this analysis the final follow-up date was December 31, 1996. Participants who emigrated where censored at migration date. Data on mortality and causes of death in the cohort-at-large were obtained from linkage with the Dutch Central Bureau of Genealogy and Statistics Netherlands. Through this linkage, 18,091 deaths were identified between January 1987 and December 1996. The completeness of the mortality follow-up was 99% [11]. Overall mortality follow-up was not available for the NLCS after this period. Causes of death were coded according to the International Classification of Disease, ninth revision (ICD-9) for 1987–1995 and ICD-10 for 1996 [12]. Besides total mortality, the following major primary causes of death were separately investigated: cancer (ICD-9: 140–239; ICD-10: C00–D48), cardiovascular (CVD) (ICD-9: 390–459; ICD-10: I00–I99), respiratory disease (ICD-9: 460–519; ICD-10: J00–J99), and all other causes excluding external injuries (ICD-9: all other codes excluding 780–799; ICD-10: all other excluding R00–R99).

### Exposure assessment

The baseline questionnaire measured dietary intake (150 items), detailed smoking habits and many other lifestyle factors, and medical conditions [9]. Habitual consumption of food and beverages during the year preceding baseline was assessed using a semi-quantitative food-frequency questionnaire, which was validated against a 9-day diet record [13]. Of the 150 food-frequency questionnaire items, 24 addressed beverage consumption. The questionnaire covered almost all beverages consumed regularly, namely water (tap or bottled), milk (i.e. milk, buttermilk, cocoa), juice (i.e. tomato, orange, others), soda and lemonade (i.e. cola, others), alcoholic beverages (i.e. beer, red wine, white wine, sherry, port, liqueur, spirits), soup, coffee, and tea. The amount of beverages consumed was measured in household units (glasses, cups, soup plates, etc.). The participants could indicate the frequency of beverage consumption and the portion size. During the validation study the capacity of the household units was measured by a dietician who visited the subjects. The average content that was established by the dietician was 175 ml for a glass, 125 ml for a cup (coffee or tea), 250 ml for a soup bowl, 200 ml for a beer glass, 105 ml for a wine glass, 80 ml for a sherry glass, and 45 ml for a liqueur/liquor glass [13]. Based on questionnaire data, the total fluid consumption was calculated using information on frequency and beverage-specific serving size of all the specific beverages. The Spearman correlation coefficient between average daily intake of non-alcoholic beverages assessed by the questionnaire and estimated from the 9-day diet record was 0.63. For alcoholic beverages, the Spearman correlation coefficient was 0.89 [13]. No validity estimates for intake of specific beverages were available. Participants were asked whether they drank coffee or tea and, if so, how many cups of each per day on average (without response categories). No questions were asked on decaffeinated coffee, as in the NLCS pilot study this item was not consumed much [14]. The type of tea was not specified but this population rarely drank any tea other than black tea [15]. For coffee, respondents could also indicate whether they drank “more, less, or the same amount” 5 years before baseline, compared to baseline. Stable coffee users were those indicating the same amount. Nutrient intakes were calculated using the computerized Dutch food composition table [16].

### Population for analysis

From the 18,091 deaths in the cohort, subjects who reported a history of cancer (excluding skin cancer) or CVD (myocardial infarction, angina pectoris, stroke) at baseline were excluded from this mortality analysis to avoid reverse causation, leaving 12,386 deaths. A similar exclusion applied to the subcohort yielded 4193 subcohort members available. (Subcohort members with prevalent cancer or CVD at baseline had more often reduced their coffee intake compared to 5 years ago, than those without CVD or cancer.) Additionally, subjects with incomplete or inconsistent dietary data were excluded, according to criteria described previously [11, 13], leaving 10,382 deaths (6701 men, 3681 women) and 3693 subcohort members (1743 men, 1950 women) available for analysis after these exclusions. Multivariate case-cohort analyses were based on 8665 deaths and 3166 subcohort members with complete data on coffee, tea and confounders. Cause-specific numbers are presented in Supplementary Figure S1.

### Statistical analysis

For the intakes of coffee and tea, the median (IQR) values were calculated in the subcohort. Associations between coffee and tea intake and various (non)dietary characteristics were examined by cross-tabulations. The relationship between intake of coffee and tea and overall mortality and cause-specific mortality was evaluated using Cox proportional hazards models; deaths due to other causes were censored at date of death for cause-specific analyses. Analyses were done for men and women separately to allow and evaluate possible effect modification by sex. The proportional hazards assumption was evaluated using − ln(− ln) survival plots, and by adding interaction terms between exposure and time to the multivariable adjusted models, and tested using Wald tests. No violation of the proportional hazards assumption was found. Standard errors were estimated using the robust Huber–White sandwich estimator to account for additional variance introduced by the subcohort sampling [17].

In age- and multivariable-adjusted survival analyses per sex, coffee and tea intake were evaluated and tested on categorical and continuous scales. In multivariable analyses, hazard ratios (HRs) were corrected for potential confounders: age at baseline (continuous, years), cigarette smoking status (coded as never, former, current smoker), number of cigarettes smoked per day, and years of smoking (both continuous, centered), history of physician-diagnosed hypertension (no, yes) and diabetes (no, yes), body height (continuous, m), BMI (< 18.5, 18.5–< 25, 25–< 30, ≥ 30 kg/m^2^), non-occupational physical activity (< 30, 30–60, 61–90, ≥ 90 min/day), highest level of education (primary school or lower vocational, secondary or medium vocational, and higher vocational or university), intake of alcohol (0, 0.1–< 5, 5–< 15, 15–< 30, 30+ g/day), vegetables and fruit (both continuous, g/day), nuts (0, 0.1–< 5, 5–< 10, 10+ g/day), energy (continuous, kcal/day), use of nutritional supplements (no, yes), and, in women, postmenopausal hormone replacement therapy (never, ever). Coffee and tea intake were mutually adjusted for each other as well. Analyses were repeated after excluding deaths occurring in the first 2 years of follow-up. Listwise deletion was applied to handle missing data for potential confounders. Tests for trends were assessed using Wald tests, by fitting median values of beverage consumption per intake category as continuous terms. Tests for non-linearity in the associations with mortality were conducted using restricted cubic splines, using three knots (10th, 50th, 90th percentiles). These survival analyses (for coffee and tea intake, respectively) were carried out for overall mortality, followed by cause-specific analyses.

The above analyses estimate associations with increasing coffee intake, while adjusting for tea intake, i.e. keeping tea intake constant, and vice versa. However, considering that many people can choose between coffee and tea as their main beverages, additional analyses were performed in which associations were estimated when substituting (replacing) tea with coffee. Standard substitution analyses assume a linear relationship between the exposure variables and disease risk/mortality [18], so that the effect of substituting equal amounts, e.g., replacing one cup of tea with one cup of coffee, can be estimated. Since the associations with coffee and tea were significantly nonlinear (see “Results”), the approach used here was by calculating the percentage tea of the total cups of coffee and tea consumed per day. This variable was categorized and used in multivariate analyses, while additionally controlling for total coffee and tea intake. This was modelled with tea percentage as exposure variable because the natural reference group of low percentage intake would be larger than with coffee percentage as exposure. Excluded from this analysis were (the very few) non-drinkers of both beverages.

To evaluate potential residual confounding by mortality risk factors, and interactions, analyses for overall mortality were also conducted in subgroups of smoking, alcohol, BMI, physical activity, and Mediterranean Diet adherence as measured with the alternate Mediterranean Diet Score (aMED) [11, 19]. Multiplicative interactions with these factors were tested using Wald tests and cross-product terms. In sensitivity analyses, we additionally adjusted for other fluid intake than coffee and tea.

All analyses were performed using Stata version 12; presented *P* values are two-sided.

## Results

Almost all participants in the NLCS consumed coffee or tea. In the subcohort, only 2.9% of men and 3.3% of women reported no coffee consumption, while for tea the percentage of nonusers was 15.5% among men and 10.8% among women. Coffee and tea consumption were the most commonly consumed beverages and accounted for 61% of total fluid consumption among men, and 66% among women. Both in subcohort men and women separately, the median (interquartile range, IQR) coffee intake among drinkers was 4 (2) cups/day [or 500 (250) ml/day, where 1 cup equals 125 ml]; for tea intake, the median (IQR) was 3 (2) cups/day, or 375 (250) ml/day among tea drinkers. Men and women who drank relatively high amounts of coffee were on average somewhat younger, while the opposite pattern was seen for tea (Table 1). Higher coffee consumption was also associated with lower fruit intake in men and women, with higher alcohol intake and BMI, and with lower nut intake in women, but higher nut intake in men. Among men and women consuming more coffee, there were fewer never smokers, Mediterranean diet adherence was lower, fewer people had university or higher vocational education, and use of nutritional supplements was lower. For tea intake, the abovementioned relationships were mostly reversed except for education. Among women, the percentage of diabetics was higher among high coffee consumers, and lower among high tea consumers. With increasing coffee intake, tea intake decreased in both sexes. The Spearman correlation coefficients between coffee and tea (cups) were − 0.26 in men and − 0.24 in women. The intake of other fluids decreased somewhat with increasing coffee intake in both sexes, and decreased more clearly with increasing tea intake in men.

**Table 1:** Baseline characteristics (means (SD), or percent) according to coffee and tea intake in male and female subcohort members with complete dietary and covariable data, Netherlands Cohort Study

Characteristic	Coffee, cups/day					Tea, cups/day			
	0–1.0 cups	1.1–< 4 cups	4–< 6 cups	≥ 6 cups		0–< 1 cups	1–< 3 cups	3–< 5 cups	≥ 5 cups
Median (cups)	1	3	4	6		0	2	4	6
Median (ml)	125	375	500	750		0	250	500	750
Men									
N	96	299	668	452		225	623	439	209
Age, mean (SD) (yr)	61.6 (4.2)	62.3 (4.2)	61.5 (4.2)	60.3 (4.0)		60.1 (3.9)	60.9 (4.1)	61.9 (4.2)	62.3 (4.3)
BMI (kg/m^2^)	24.3 (3.1)	24.9 (2.5)	24.9 (2.4)	24.9 (2.7)		25.1 (2.6)	24.8 (2.6)	25.0 (2.5)	24.6 (2.4)
Physical activity, nonoccupational (min/day)	81.4 (77.9)	82.1 (66.1)	79.6 (65.8)	80.3 (71.5)		82 (79.3)	77.1 (62.1)	80.6 (66.6)	86.8 (77.7)
Alcohol intake (g/day)	9.5 (17.5)	14.1 (15.4)	15.9 (17.0)	16.0 (18.1)		17.6 (21.0)	16.5 (16.9)	14.0 (15.8)	11.1 (13.3)
Vegetable intake (g/day)	188.8 (87.1)	188.7 (74.2)	182.8 (70.7)	188 (80.3)		183.3 (83)	182.1 (72.5)	189.1 (75)	192.2 (72.3)
Fruit intake (g/day)	190.5 (176.1)	165.1 (121.9)	152.8 (101.3)	140 (110.7)		135 (114.4)	147 (112.2)	163.5 (108.9)	173.7 (135.3)
Nut intake (g/day)	6.8 (15.3)	7.6 (13.8)	7.8 (12.6)	9 (16)		8.6 (18.1)	9.1 (15.8)	7.5 (11.8)	6.1 (10.3)
Tea intake (cups/day)	3.7 (2.4)	3.0 (1.9)	2.7 (1.9)	2.0 (1.8)	Coffee	5.9 (3.1)	4.7 (1.9)	4.3 (1.9)	3.8 (2.4)
Other fluids intake (ml/day)	606.4 (436.4)	569.4 (303.6)	548.8 (323.2)	569.2 (363.2)		663.9 (401.1)	569 (332.8)	526 (314.3)	523.8 (323.8)
Never smoker (%)	40.6	17.7	12.3	10.0		12.4	12.5	17.1	16.3
Low MD-adherence (aMED 0–3 pts) (%)	30.2	29.4	31.1	36.1		40.4	31.0	31.0	29.7
University or higher vocational education (%)	20.8	27.4	21.4	17.3		20.4	20.9	22.6	22.0
Diabetes (%)	2.1	4.0	2.5	3.1		4.4	1.9	3.6	2.4
Hypertension (%)	19.8	32.8	20.5	18.4		19.1	25.4	21.0	21.5
Nutritional supplement user (%)	34.4	30.1	21.9	18.8		23.6	22.0	22.8	30.1
Women									
N	114	490	723	324		163	555	571	328
Age, mean (SD) (year)	62.1 (4.5)	62.2 (4.2)	61.4 (4.2)	60.7 (4.0)		61.2 (4.4)	60.9 (4.1)	61.9 (4.2)	62.1 (4.1)
BMI (kg/m^2^)	24.1 (3.7)	24.6 (3.3)	25.2 (3.5)	25.5 (3.7)		25.4 (4.0)	25.3 (3.6)	25.0 (3.5)	24.4 (3.2)
Physical activity, nonoccupational (min/day)	66 (45.2)	65.9 (51.9)	65.4 (52.7)	64.1 (50.5)		62.5 (49.9)	66.5 (53.7)	63 (50.8)	68.9 (50.2)
Alcohol intake (g/day)	4.4 (9.5)	6.4 (9.8)	6.6 (10.1)	5.3 (7.7)		6.4 (9.6)	6.3 (10.2)	5.9 (9.1)	6.0 (9.3)
Vegetable intake (g/day)	203.3 (91)	183.4 (66.8)	193.8 (72.8)	196.3 (83.1)		189.9 (86.6)	184.5 (73.1)	197.7 (70.4)	195 (77)
Fruit intake (g/day)	215.3 (117.6)	194.2 (119.1)	197.5 (113.1)	179.1 (128.9)		161.1 (113.4)	191.7 (117.3)	198.2 (115)	209 (128.4)
Nut intake (g/day)	5.2 (13.6)	4.4 (8.7)	4.4 (7.7)	4.4 (8.2)		3.9 (8)	4.3 (8.8)	4.8 (8.7)	4.5 (8.8)
Tea intake (cups/day)	4.6 (2.7)	3.4 (2.1)	3.0 (1.8)	2.4 (2.0)	Coffee	5.1 (2.4)	4.2 (1.8)	3.9 (1.7)	3.3 (1.9)
Other fluids intake (ml/day)	473.7 (280.2)	479.4 (297.3)	466.1 (274.9)	420.1 (280.2)		477.5 (357.8)	464.6 (276.1)	466.3 (278.1)	439.8 (262)
Never smoker (%)	71.9	65.3	59.3	44.1		44.2	56.2	62.0	65.5
Low MD-adherence (aMED 0–3 pts) (%)	26.3	31.2	31.5	35.5		42.9	32.4	28.9	29.9
University or higher vocational education (%)	15.8	15.5	8.0	5.6		12.3	6.8	9.1	16.2
Diabetes (%)	0.9	3.1	2.8	4.3		4.3	3.1	3.0	1.8
Hypertension (%)	24.6	30.2	28.4	21.0		25.2	24.1	32.2	24.4
Nutritional supplement user (%)	54.4	41.4	34.7	33.3		33.1	38.4	38.2	38.1
Ever used hormone replacement therapy (%)	12.3	13.3	14.4	11.4		9.2	12.8	15.1	12.5

**Fig. 1:**
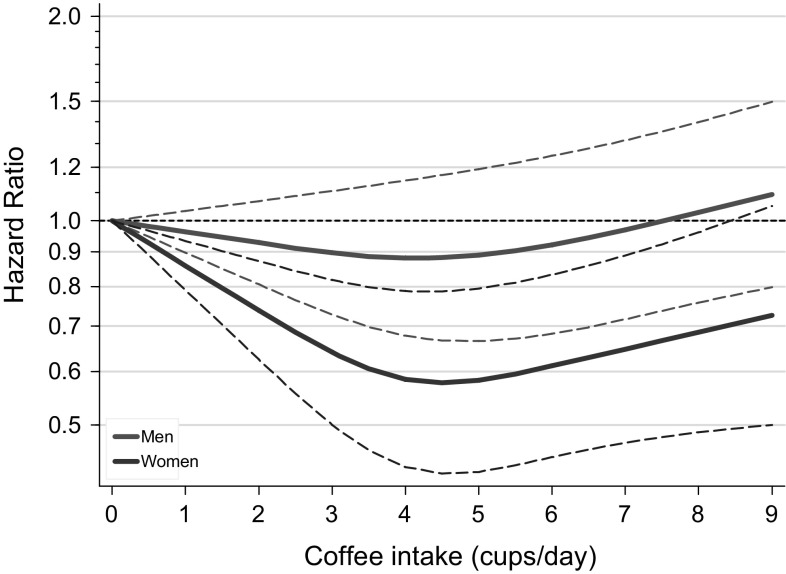
Spline regression curves for the association between coffee intake and total mortality. Red lines: men. Blue lines: women. Solid lines represents point estimates and dashed lines represent 95% CI. Multivariate HRs are calculated by restricted cubic spline regression (using 3 knots at 10th, 50th, and 90th percentiles) adjusting for: age at baseline (continuous, in years), cigarette smoking status (coded as never, former, current smoker), number of cigarettes smoked per day, and years of smoking (both continuous, centered), history of physician-diagnosed hypertension (no, yes) and diabetes (no, yes), body height (continuous, m), BMI (< 18.5, 18.5–< 25, 25–< 30, ≥ 30 kg/m^2^), non-occupational physical activity (< 30, 30–60, 61–90, ≥ 90 min/day), highest level of education (primary school or lower vocational, secondary or medium vocational, and higher vocational or university), intake of alcohol (0, 0.1–< 5, 5–< 15, 15–< 30, 30+ g/day), nuts (0, 0.1–< 5, 5–< 10, 10+ g/day), vegetables and fruit (both continuous, g/day), tea (continuous, cups/day), energy (continuous, kcal/day), use of nutritional supplements (no, yes), and, in women, postmenopausal HRT (never, ever). (Color figure online)

Of the 8665 deaths with complete information on coffee and tea intake and potential confounders, 5636 occurred in men and 3029 in women. Age- and multivariable-adjusted analyses of coffee intake and mortality showed significant heterogeneity between men and women (*P* = 0.018); subsequent analyses were therefore done per sex. While there was a statistically significant positive association between coffee intake and overall mortality in men in age-adjusted analyses (Table 2), no clear association was observed after multivariable adjustment, with a HR (95% CI) of 1.22 (0.89–1.66) when comparing 6+ versus 0–1 cups/day (*P*_trend_ = 0.320). In women, coffee intake was significantly inversely related to overall mortality after multivariable adjustment, with a HR (95% CI) of 0.65 (0.47–0.90) when comparing 6+ versus 0–1 cups/day (*P*_trend_ = 0.014). Analyses excluding the first 2 years of follow-up showed similar results (data not shown). Restricted cubic splines (Fig. 1) showed deviations from linearity between coffee and mortality, especially in women (Table 2, *P* for nonlinearity = 0.055 in men, *P* = 0.001 in women).

**Table 2:** Overall and cause-specific mortality according to coffee intake in multivariable-adjusted^a^ analyses, NLCS

	Coffee (cups/day) (median)						*P* trend	*P* non-linearity
	0–1.0 cups/d	1.1–< 3 cups/d	3–< 4 cups/d	4–< 5 cups/d	5–< 6 cups/d	6+ cups/d		
	(1)	(2)	(3)	(4)	(5)	(6)		
All causes								
Men								
No. of deaths	295	541	687	1413	735	1965		
Person-years in subcohort	862	1326	1353	3796	2373	4077		
Age-adjusted HR	1 (Ref)	1.03	1.38	1.03	0.94	1.60	< 0.001	
(95% CI)		(0.75–1.41)	(1.01–1.90)	(0.78–1.36)	(0.70–1.26)	(1.22–2.11)		
Multivariable-adjusted HR	1 (Ref)	0.95	1.22	0.90	0.74	1.22	0.320	0.055
(95% CI)		(0.67–1.33)	(0.86–1.73)	(0.66–1.22)	(0.53–1.03)	(0.89–1.66)		
Women								
No. of deaths	279	458	476	875	335	606		
Person-years in subcohort	1089	2171	2535	5142	2001	3169		
Age-adjusted HR	1 (Ref)	0.80	0.74	0.70	0.72	0.87	0.511	
(95% CI)		(0.61–1.06)	(0.56–0.98)	(0.54–0.91)	(0.54–0.96)	(0.66–1.14)		
Multivariable-adjusted HR	1 (Ref)	0.74	0.70	0.63	0.59	0.65	0.014	0.001
(95% CI)		(0.54–1.01)	(0.51–0.96)	(0.47–0.84)	(0.42–0.83)	(0.47–0.90)		
Cancer								
Men								
No. of deaths	101	207	280	602	338	901		
Age-adjusted HR	1 (Ref)	1.16	1.66	1.29	1.26	2.13	< 0.001	
(95% CI)		(0.81–1.68)	(1.16–2.38)	(0.94–1.77)	(0.90–1.76)	(1.55–2.92)		
Multivariable-adjusted HR	1 (Ref)	1.10	1.48	1.06	0.95	1.49	0.060	0.433
(95% CI)		(0.74–1.65)	(0.99–2.19)	(0.75–1.52)	(0.65–1.38)	(1.04–2.13)		
Women								
No. of deaths	117	208	207	431	160	297		
Age-adjusted HR	1 (Ref)	0.87	0.76	0.81	0.79	0.96	0.832	
(95% CI)		(0.63–1.21)	(0.55–1.05)	(0.60–1.08)	(0.57–1.11)	(0.71–1.31)		
Multivariable-adjusted HR	1 (Ref)	0.81	0.70	0.74	0.65	0.75	0.201	0.008
(95% CI)		(0.57–1.14)	(0.49–0.99)	(0.53–1.03)	(0.44–0.95)	(0.52–1.08)		
Cardiovascular disease								
Men								
No. of deaths	111	207	248	479	255	695		
Age-adjusted HR	1 (Ref)	1.03	1.32	0.92	0.87	1.53	0.004	
(95% CI)		(0.71–1.49)	(0.91–1.91)	(0.66–1.28)	(0.61–1.23)	(1.11–2.12)		
Multivariable-adjusted HR	1 (Ref)	0.86	1.08	0.80	0.69	1.22	0.155	0.073
(95% CI)		(0.57–1.28)	(0.71–1.63)	(0.56–1.15)	(0.47–1.03)	(0.84–1.76)		
Women								
No. of deaths	87	150	161	248	96	190		
Age-adjusted HR	1 (Ref)	0.84	0.81	0.66	0.69	0.93	0.562	
(95% CI)		(0.58–1.20)	(0.56–1.16)	(0.47–0.92)	(0.47–1.02)	(0.65–1.32)		
Multivariable-adjusted HR	1 (Ref)	0.80	0.81	0.61	0.58	0.72	0.079	0.015
(95% CI)		(0.52–1.23)	(0.52–1.26)	(0.41–0.93)	(0.36–0.95)	(0.46–1.14)		
Respiratory disease								
Men								
No. of deaths	26	45	69	105	37	111		
Age-adjusted HR	1 (Ref)	0.91	1.52	0.84	0.53	1.07	0.419	
(95% CI)		(0.51–1.61)	(0.88–2.62)	(0.51–1.40)	(0.30–0.95)	(0.65–1.77)		
Multivariable-adjusted HR	1 (Ref)	0.86	1.55	0.62	0.35	0.69	0.006	0.023
(95% CI)		(0.45–1.65)	(0.82–2.93)	(0.34–1.11)	(0.18–0.69)	(0.38–1.25)		
Women								
No. of deaths	12	16	12	49	19	30		
Age-adjusted HR	1 (Ref)	0.65	0.43	0.93	0.97	1.04	0.134	
(95% CI)		(0.30–1.43)	(0.19–1.01)	(0.47–1.82)	(0.45–2.08)	(0.51–2.11)		
Multivariable-adjusted HR	1 (Ref)	0.70	0.56	0.91	0.77	0.77	0.918	0.725
(95% CI)		(0.25–1.94)	(0.21–1.51)	(0.37–2.22)	(0.29–2.07)	(0.30–2.02)		
Other causes								
Men								
No. of deaths	50	71	77	197	91	220		
Age-adjusted HR	1 (Ref)	0.81	0.93	0.85	0.68	1.04	0.561	
(95% CI)		(0.51–1.28)	(0.59–1.45)	(0.58–1.26)	(0.44–1.05)	(0.71–1.54)		
Multivariable-adjusted HR	1 (Ref)	0.85	0.89	0.90	0.59	0.92	0.541	0.022
(95% CI)		(0.52–1.40)	(0.54–1.47)	(0.58–1.39)	(0.36–0.96)	(0.59–1.43)		
Women								
No. of deaths	56	75	85	122	49	75		
Age-adjusted HR	1 (Ref)	0.65	0.66	0.49	0.53	0.55	0.006	
(95% CI)		(0.43–1.00)	(0.44–1.00)	(0.33–0.73)	(0.34–0.84)	(0.36–0.84)		
Multivariable-adjusted HR	1 (Ref)	0.50	0.53	0.33	0.39	0.30	< 0.001	0.001
(95% CI)		(0.31–0.81)	(0.32–0.86)	(0.20–0.53)	(0.23–0.67)	(0.17–0.52)		

^a^Multivariable analyses were adjusted for: age at baseline (continuous, in years), cigarette smoking status (coded as never, former, current smoker), number of cigarettes smoked per day, and years of smoking (both continuous, centered)), history of physician-diagnosed hypertension (no, yes) and diabetes (no, yes), body height (continuous, m), BMI (< 18.5, 18.5–< 25, 25–< 30, ≥ 30 kg/m^2^), non-occupational physical activity (< 30, 30–60, 61–90, ≥ 90 min/day), highest level of education (primary school or lower vocational, secondary or medium vocational, and higher vocational or university), intake of alcohol (0, 0.1–< 5, 5–< 15, 15–< 30, 30+ g/day), nuts (0, 0.1–< 5, 5–< 10, 10+ g/day), vegetables and fruit (both continuous, g/day), tea (continuous, cups/day), energy (continuous, kcal/day), use of nutritional supplements (no, yes), and, in women, postmenopausal HRT (never, ever)

In multivariable analyses of major causes of death per sex (Table 2), coffee intake was borderline significantly positively associated with death due to cancer in men, with a HR (95% CI) of 1.49 (1.04–2.13) when comparing 6+ versus 0–1 cups/day (*P*_trend_ = 0.060). However, an inverse association was seen in women with significantly decreased HRs in several intake categories, such as a HR (95% CI) of 0.65 (0.44–0.95) when comparing 5–< 6 versus 0–1 cups/day. There was clear evidence for a nonlinear association in women (*P* for nonlinearity = 0.008). A comparable pattern was seen for CVD deaths. For death due to respiratory disease, a significant inverse association with coffee intake was seen in men (*P*_trend_ = 0.006), while there was no significant association in women. For deaths due to other causes, there was evidence for nonlinear inverse associations with coffee intake in both men and women. Supplementary Figure S2 shows cause-specific nonparametric regression curves from these analyses, in men and women. The percentage of stable coffee users in the subcohort was 74% in men and 78% in women. Sensitivity analyses limited to those with stable coffee intake (with 4182 deaths in men and 2235 in women) showed essentially similar patterns to the whole population (Supplementary Figure S3).

**Fig. 2:**
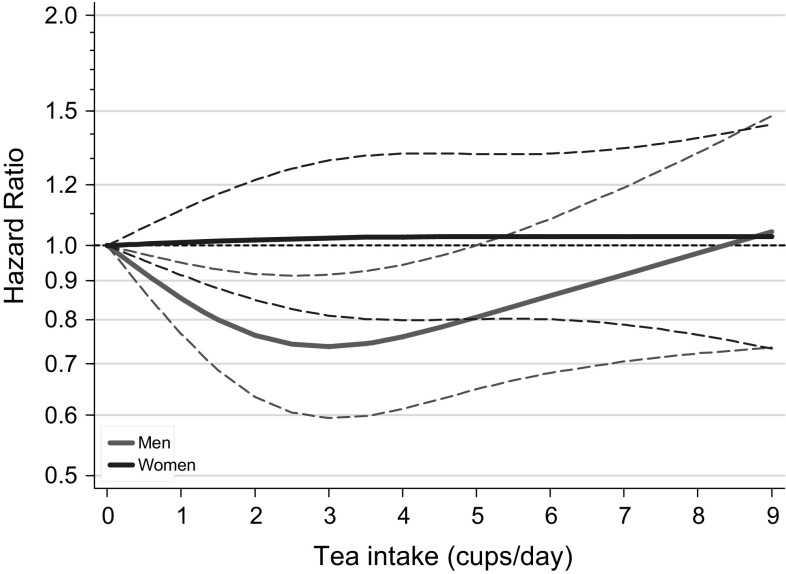
Spline regression curves for the association between tea intake and total mortality. Red lines: men. Blue lines: women. Multivariate HRs are calculated by restricted cubic spline regression (using 3 knots at 10th, 50th, and 90th percentiles) adjusting for: age at baseline (continuous, in years), cigarette smoking status (coded as never, former, current smoker), number of cigarettes smoked per day, and years of smoking (both continuous, centered), history of physician-diagnosed hypertension (no, yes) and diabetes (no, yes), body height (continuous, m), BMI (< 18.5, 18.5–< 25, 25–< 30, ≥ 30 kg/m^2^), non-occupational physical activity (< 30, 30–60, 61–90, ≥ 90 min/day), highest level of education (primary school or lower vocational, secondary or medium vocational, and higher vocational or university), intake of alcohol (0, 0.1–< 5, 5–< 15, 15–< 30, 30+ g/day), nuts (0, 0.1–< 5, 5–< 10, 10+ g/day), vegetables and fruit (both continuous, g/day), coffee (continuous, cups/day), energy (continuous, kcal/day), use of nutritional supplements (no, yes), and, in women, postmenopausal HRT (never, ever). (Color figure online)

Tea intake showed a nonlinear inverse association with overall mortality in men *P* for nonlinearity = 0.004), but no association in women, after multivariable adjustment (Table 3; Fig. 2). The lowest significantly decreased HR (0.72, 95% CI 0.57–0.91) was observed in men drinking 2–3 cups/day, compared to nonconsumers. As in the analyses for coffee, there was a large effect of multivariable adjustment on the risk estimates, compared to age-adjusted analyses. In cause-specific analyses for tea, deaths due to cancer and CVD were significantly inversely related to tea intake in men in a nonlinear fashion (Table 3 and Supplementary Figure S4), while there was no association with deaths due to respiratory or other causes. In women, tea intake did not show clear associations with cause-specific mortality.

**Table 3:** Overall and cause-specific mortality according to tea intake in multivariable-adjusted^a^ analyses, NLCS

	Tea (cups/day) (median)						*P* trend	*P* non-linearity
	0	0.1–< 2	2–< 3	3–< 4	4–< 5	5+		
	(0)	(1)	(2)	(3)	(4)	(6)		
All causes								
Men								
No. of deaths	1030	815	1384	702	931	774		
Person-years in subcohort	1924	2205	3631	1729	2373	1925		
Age-adjusted HR	1 (Ref)	0.72	0.64	0.65	0.57	0.59	< 0.001	
(95% CI)		(0.57–0.91)	(0.52–0.78)	(0.51–0.84)	(0.45–0.72)	(0.46–0.75)		
Multivariable-adjusted HR	1 (Ref)	0.81	0.72	0.83	0.76	0.77	0.084	0.004
(95% CI)		(0.63–1.04)	(0.57–0.91)	(0.64–1.09)	(0.58–0.98)	(0.58–1.01)		
Women								
No. of deaths	365	302	670	425	656	611		
Person-years in subcohort	1510	1869	3718	2389	3372	3249		
Age-adjusted HR	1 (Ref)	0.71	0.78	0.70	0.77	0.74	0.083	
(95% CI)		(0.54–0.94)	(0.62–1.00)	(0.54–0.91)	(0.60–0.98)	(0.58–0.94)		
Multivariable-adjusted HR	1 (Ref)	0.78	0.94	0.87	0.94	0.97	0.697	0.905
(95% CI)		(0.57–1.07)	(0.72–1.24)	(0.64–1.17)	(0.70–1.26)	(0.72–1.30)		
Cancer								
Men								
Person-years in subcohort	1924	2205	3631	1729	2373	1925		
No. of deaths	462	344	589	289	404	341		
Age-adjusted HR	1 (Ref)	0.67	0.61	0.61	0.57	0.60	< 0.001	
(95% CI)		(0.53–0.86)	(0.49–0.76)	(0.47–0.80)	(0.44–0.73)	(0.46–0.77)		
Multivariable-adjusted HR	1 (Ref)	0.75	0.69	0.77	0.77	0.79	0.243	0.004
(95% CI)		(0.57–0.98)	(0.54–0.89)	(0.57–1.03)	(0.58–1.01)	(0.59–1.06)		
Women								
Person-years in subcohort	1510	1869	3718	2389	3372	3249		
No. of deaths	159	144	326	203	304	284		
Age-adjusted HR	1 (Ref)	0.76	0.85	0.78	0.82	0.80	0.276	
(95% CI)		(0.55–1.04)	(0.65–1.12)	(0.58–1.04)	(0.63–1.08)	(0.60–1.05)		
Multivariable-adjusted HR	1 (Ref)	0.79	0.99	0.91	0.99	0.98	0.579	0.651
(95% CI)		(0.56–1.11)	(0.74–1.33)	(0.66–1.26)	(0.73–1.35)	(0.72–1.35)		
Cardiovascular disease								
Men								
No. of deaths	363	298	472	248	334	280		
Age-adjusted HR	1 (Ref)	0.75	0.61	0.65	0.56	0.59	< 0.001	
(95% CI)		(0.58–0.98)	(0.47–0.77)	(0.49–0.86)	(0.43–0.73)	(0.45–0.78)		
Multivariable-adjusted HR	1 (Ref)	0.83	0.66	0.81	0.69	0.72	0.040	0.003
(95% CI)		(0.61–1.12)	(0.51–0.87)	(0.59–1.10)	(0.51–0.94)	(0.52–0.98)		
Women								
No. of deaths	123	89	187	136	197	200		
Age-adjusted HR	1 (Ref)	0.65	0.67	0.67	0.68	0.71	0.195	
(95% CI)		(0.45–0.93)	(0.49–0.92)	(0.48–0.94)	(0.50–0.93)	(0.52–0.98)		
Multivariable-adjusted HR	1 (Ref)	0.84	0.97	0.97	0.94	1.19	0.295	0.800
(95% CI)		(0.54–1.32)	(0.66–1.42)	(0.64–1.47)	(0.62–1.42)	(0.80–1.78)		
Respiratory disease								
Men								
No. of deaths	69	54	110	57	59	44		
Age-adjusted HR	1 (Ref)	0.74	0.71	0.74	0.48	0.45	< 0.001	
(95% CI)		(0.48–1.13)	(0.49–1.04)	(0.48–1.14)	(0.31–0.74)	(0.28–0.71)		
Multivariable-adjusted HR	1 (Ref)	0.67	0.84	1.09	0.81	0.61	0.308	0.920
(95% CI)		(0.38–1.17)	(0.53–1.32)	(0.65–1.82)	(0.49–1.33)	(0.35–1.07)		
Women								
No. of deaths	18	15	37	8	32	28		
Age-adjusted HR	1 (Ref)	0.73	0.89	0.27	0.75	0.68	0.227	
(95% CI)		(0.36–1.51)	(0.49–1.62)	(0.11–0.63)	(0.41–1.39)	(0.36–1.27)		
Multivariable-adjusted HR	1 (Ref)	0.86	1.08	0.39	1.02	0.97	0.971	0.607
(95% CI)		(0.38–1.95)	(0.53–2.21)	(0.15–1.05)	(0.47–2.22)	(0.42–2.23)		
Other causes								
Men								
No. of deaths	119	103	184	93	114	93		
Age-adjusted HR	1 (Ref)	0.78	0.74	0.76	0.62	0.63	0.004	
(95% CI)		(0.56–1.09)	(0.55–1.00)	(0.54–1.08)	(0.44–0.86)	(0.45–0.89)		
Multivariable-adjusted HR	1 (Ref)	1.06	0.98	1.06	0.91	0.92	0.483	0.358
(95% CI)		(0.71–1.60)	(0.69–1.41)	(0.70–1.59)	(0.61–1.34)	(0.60–1.38)		
Women								
No. of deaths	55	47	108	64	106	82		
Age-adjusted HR	1 (Ref)	0.75	0.85	0.70	0.82	0.65	0.080	
(95% CI)		(0.48–1.18)	(0.58–1.26)	(0.46–1.07)	(0.55–1.20)	(0.44–0.97)		
Multivariable-adjusted HR	1 (Ref)	0.72	0.88	0.67	0.80	0.68	0.192	0.999
(95% CI)		(0.43–1.23)	(0.55–1.41)	(0.40–1.13)	(0.49–1.30)	(0.41–1.11)		

^a^Multivariable analyses were adjusted for: age at baseline (continuous, in years), cigarette smoking status (coded as never, former, current smoker), number of cigarettes smoked per day, and years of smoking (both continuous, centered)), history of physician-diagnosed hypertension (no, yes) and diabetes (no, yes), body height (continuous, m), BMI (< 18.5, 18.5–< 25, 25–< 30, ≥ 30 kg/m^2^), non-occupational physical activity (< 30, 30–60, 61–90, ≥ 90 min/day), highest level of education (primary school or lower vocational, secondary or medium vocational, and higher vocational or university), intake of alcohol (0, 0.1–< 5, 5–< 15, 15–< 30, 30+ g/day), nuts (0, 0.1–< 5, 5–< 10, 10+ g/day), vegetables and fruit (both continuous, g/day), coffee (continuous, cups/day), energy (continuous, kcal/day), use of nutritional supplements (no, yes), and, in women, postmenopausal HRT (never, ever)

**Fig. 3:**
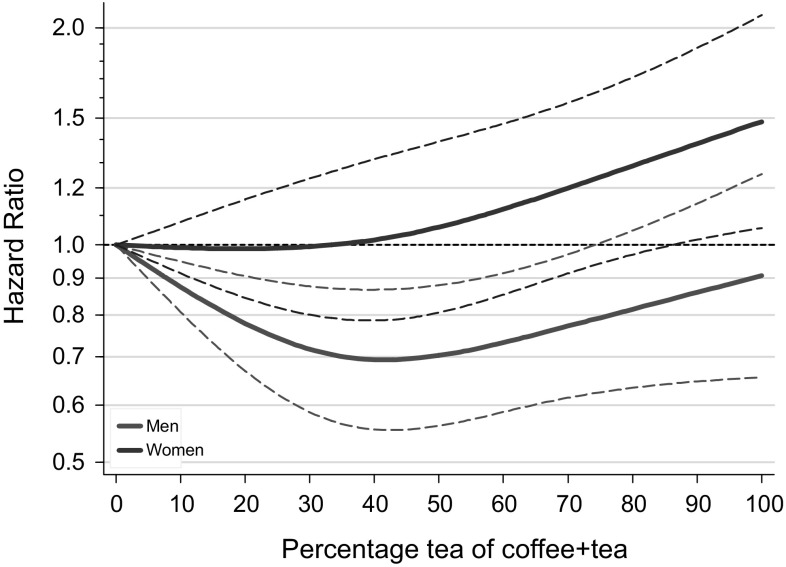
Spline regression curves for the association between percentage tea of total coffee and tea and total mortality in substitution analyses. Red lines: men. Blue lines: women. Multivariate HRs are calculated by restricted cubic spline regression (using 3 knots at 10th, 50th, and 90th percentiles) adjusting for: age at baseline (continuous, in years), cigarette smoking status (coded as never, former, current smoker), number of cigarettes smoked per day, and years of smoking (both continuous, centered), history of physician-diagnosed hypertension (no, yes) and diabetes (no, yes), body height (continuous, m), BMI (< 18.5, 18.5–< 25, 25–< 30, ≥ 30 kg/m^2^), non-occupational physical activity (< 30, 30–60, 61–90, ≥ 90 min/day), highest level of education (primary school or lower vocational, secondary or medium vocational, and higher vocational or university), intake of alcohol (0, 0.1–< 5, 5–< 15, 15–< 30, 30+ g/day), nuts (0, 0.1–< 5, 5–< 10, 10+ g/day), vegetables and fruit (both continuous, g/day), coffee + tea (continuous, cups/day), energy (continuous, kcal/day), use of nutritional supplements (no, yes), and, in women, postmenopausal HRT (never, ever). (Color figure online)

In the substitution analyses addressing the question “Coffee or Tea?”, the percentage tea of total coffee and tea intake showed a significantly inverse association with overall mortality in men which was nonlinear, and a positive association in women, after multivariable adjustment (Table 4, and Fig. 3). In men, hazard ratios were below one for any nonzero percentage tea drinking, compared to exclusive coffee drinkers; the lowest hazard ratio was observed when the percentage tea intake was 30–50% (Fig. 3). In women, however, the lowest mortality risk was observed for those drinking less than 40% tea, and the HR increased with higher tea percentages to significantly elevated levels of 1.41 (1.01–1.99) when comparing 80–100% tea drinkers to 0–< 20% tea drinkers. The HR per increment of 10% tea was 1.04 (1.00–1.07) (there was no evidence for nonlinearity in women). In cause-specific analyses of percentage tea intake, both deaths due  to cancer and CVD were significantly inversely related to percentage tea intake in men in a nonlinear fashion (Table 4 and Supplementary Figure S5), while there was no significant association with deaths due to respiratory or other causes of death. In women, percentage tea intake showed significantly positive associations with deaths due to CVD and Other causes (Table 4, Figure S5).

**Table 4:** Mortality according to tea intake as percentage of coffee and tea combined, in multivariable-adjusted^a^ substitution analyses, NLCS

	Percentage tea of coffee + tea cups/day (median)					*P* trend	*P* non-linearity
	0–< 20%	20–< 40%	40–< 60%	60–< 80%	80–100%		
	(0)	(28.6)	(50.0)	(66.7)	(92.0)		
Men							
All causes							
No. of deaths	1509	1721	1615	547	228		
Person-years in subcohort	3222	4714	3813	1365	617		
Age-adjusted HR	1 (Ref)	0.68	0.67	0.61	0.65	< 0.001	
(95% CI)		(0.57–0.81)	(0.56–0.80)	(0.48–0.78)	(0.47–0.90)		
Multivariable-adjusted HR	1 (Ref)	0.74	0.82	0.84	0.93	0.353	0.004
(95% CI)		(0.62–0.90)	(0.67–1.01)	(0.64–1.10)	(0.66–1.32)		
Cancer							
No. of deaths	663	772	694	214	83		
Age-adjusted HR	1 (Ref)	0.70	0.68	0.57	0.55	< 0.001	
(95% CI)		(0.59–0.85)	(0.55–0.82)	(0.43–0.74)	(0.38–0.80)		
Multivariable-adjusted HR	1 (Ref)	0.75	0.84	0.77	0.85	0.177	0.007
(95% CI)		(0.61–0.92)	(0.67–1.05)	(0.57–1.05)	(0.57–1.27)		
Cardiovascular disease							
No. of deaths	544	570	585	207	83		
Age-adjusted HR	1 (Ref)	0.61	0.65	0.62	0.65	0.001	
(95% CI)		(0.50–0.75)	(0.52–0.80)	(0.47–0.82)	(0.44–0.94)		
Multivariable-adjusted HR	1 (Ref)	0.67	0.73	0.77	0.89	0.179	0.001
(95% CI)		(0.54–0.84)	(0.57–0.93)	(0.57–1.06)	(0.59–1.34)		
Respiratory disease							
No. of deaths	88	123	121	42	16		
Age-adjusted HR	1 (Ref)	0.78	0.75	0.70	0.73	0.116	
(95% CI)		(0.56–1.09)	(0.53–1.06)	(0.45–1.10)	(0.39–1.36)		
Multivariable-adjusted HR	1 (Ref)	0.99	1.52	1.41	1.27	0.074	0.896
(95% CI)		(0.65–1.50)	(0.98–2.36)	(0.77–2.57)	(0.63–2.57)		
Other causes							
No. of deaths	189	216	181	76	42		
Age-adjusted HR	1 (Ref)	0.69	0.62	0.71	0.98	0.114	
(95% CI)		(0.54–0.89)	(0.47–0.81)	(0.50–1.00)	(0.63–1.52)		
Multivariable-adjusted HR	1 (Ref)	0.82	0.80	1.06	1.25	0.613	0.123
(95% CI)		(0.62–1.10)	(0.58–1.10)	(0.71–1.57)	(0.77–2.04)		
Women							
All causes							
No. of deaths	483	672	1035	473	229		
Person-years in subcohort	2182	4340	5362	2568	949		
Age-adjusted HR	1 (Ref)	0.72	0.82	0.75	0.97	0.581	
(95% CI)		(0.58–0.89)	(0.68–1.01)	(0.59–0.94)	(0.72–1.31)		
Multivariable-adjusted HR	1 (Ref)	0.91	1.11	1.03	1.41	0.054	0.229
(95% CI)		(0.72–1.15)	(0.87–1.41)	(0.77–1.37)	(1.01–1.99)		
Cancer							
No. of deaths	223	328	495	208	101		
Age-adjusted HR	1 (Ref)	0.75	0.86	0.73	0.96	0.484	
(95% CI)		(0.59–0.95)	(0.69–1.08)	(0.56–0.95)	(0.68–1.35)		
Multivariable-adjusted HR	1 (Ref)	0.88	1.06	0.92	1.24	0.342	0.593
(95% CI)		(0.68–1.13)	(0.82–1.37)	(0.68–1.25)	(0.84–1.81)		
Cardiovascular disease							
No. of deaths	156	189	315	171	66		
Age-adjusted HR	1 (Ref)	0.64	0.77	0.81	0.84	0.794	
(95% CI)		(0.48–0.85)	(0.59–1.00)	(0.60–1.09)	(0.57–1.24)		
Multivariable-adjusted HR	1 (Ref)	0.92	1.12	1.28	1.40	0.042	0.229
(95% CI)		(0.66–1.29)	(0.80–1.57)	(0.87–1.88)	(0.87–2.25)		
Respiratory disease							
No. of deaths	24	35	43	16	10		
Age-adjusted HR	1 (Ref)	0.76	0.68	0.50	0.84	0.154	
(95% CI)		(0.44–1.31)	(0.40–1.15)	(0.26–0.96)	(0.38–1.84)		
Multivariable-adjusted HR	1 (Ref)	1.09	1.19	0.62	1.62	0.912	0.791
(95% CI)		(0.55–2.15)	(0.60–2.36)	(0.25–1.54)	(0.60–4.34)		
Other causes							
No. of deaths	67	105	157	64	47		
Age-adjusted HR	1 (Ref)	0.82	0.89	0.71	1.41	0.643	
(95% CI)		(0.57–1.16)	(0.64–1.25)	(0.48–1.06)	(0.89–2.21)		
Multivariable-adjusted HR	1 (Ref)	1.11	1.34	1.07	2.44	0.023	0.356
(95% CI)		(0.72–1.69)	(0.87–2.07)	(0.65–1.77)	(1.39–4.28)		

^a^Multivariable analyses were adjusted for: age at baseline (continuous, in years), cigarette smoking status (coded as never, former, current smoker), number of cigarettes smoked per day, and years of smoking (both continuous, centered)), history of physician-diagnosed hypertension (no, yes) and diabetes (no, yes), body height (continuous, m), BMI (< 18.5, 18.5–< 25, 25–< 30, ≥ 30 kg/m^2^), non-occupational physical activity (< 30, 30–60, 61–90, ≥ 90 min/day), highest level of education (primary school or lower vocational, secondary or medium vocational, and higher vocational or university), intake of alcohol (0, 0.1–< 5, 5–< 15, 15–< 30, 30+ g/day), nuts (0, 0.1–< 5, 5–< 10, 10+ g/day), vegetables and fruit (both continuous, g/day), coffee + tea (continuous, cups/day), energy (continuous, kcal/day), use of nutritional supplements (no, yes), and, in women, postmenopausal HRT (never, ever)

There was no significant interaction between coffee and tea drinking in relation to total mortality among men (*P*_interaction_ = 0.908) nor women (*P*_interaction_ = 0.422). Supplementary Table 1 shows HRs for combined categories of coffee and tea intake in men and women, respectively. Compared to those drinking 5+ cups coffee/day and 0–< 2 cups of tea, HRs in other categories (increasing tea and decreasing coffee) were decreased in men, and generally increased in women. The lowest mortality HR [0.74 (0.45–1.22)] in men was observed for those drinking 0–< 2 cups coffee/day and 2–< 4 cups of tea. Women drinking 0–< 2 cups coffee/day and 2–< 4 cups of tea had a significantly elevated HR of 1.92 (1.06–3.45) compared to women drinking 5+ cups coffee/day and 0–< 2 cups of tea. The lowest mortality HRs in women were observed for those drinking relatively high coffee and low tea quantities. Cause-specific analyses of interaction between coffee and tea were not significant (data not shown).

In subgroup analyses of percentage tea intake (categorized as above) and overall mortality within categories of smoking, alcohol, BMI, physical activity, and MD-adherence, only BMI showed a significant interaction with percentage tea intake in women (*P*_interaction_ = 0.011) and not in men (*P*_interaction_ = 0.599). This is further illustrated in Supplementary Figure S6, where a strong positive association with percentage tea intake is visible in overweight women and no association in normal weight women. Because the percentage never smokers was very low in NLCS men, smoking was categorized into a subgroup of never smokers or those who stopped ≥ 10 years ago, and a subgroup of current smokers or stopped < 10 years ago. Both subgroups showed comparable associations between percentage tea intake and mortality, in men (*P*_interaction_ = 0.954) and women (*P*_interaction_ = 0.541) (Supplementary Figure S7). Additional adjustment for other fluid intake in sensitivity analyses yielded essentially similar estimates (data not shown).

## Discussion

In this large prospective study higher coffee intake was significantly and nonlinearly related to lower overall mortality in women, after adjusting for confounders including tea intake. When comparing 6+ versus 0–1 cups (of 125 ml) coffee/day, the HR (95% CI) was 0.65 (0.47–0.90). No significant association was seen with overall mortality in men. In women, the inverse association with coffee was seen for cancer, cardiovascular mortality and other causes of death, but not for respiratory mortality, while the results for men showed the opposite. Restricted cubic splines analyses indicated that drinking up to 5 cups (or 625 ml)/day of coffee was associated with significantly decreased mortality in women, with no further decrease in mortality with higher intakes. In contrast, tea intake was significantly nonlinearly related to lower overall, cancer and CVD mortality in men, but showed no association with overall mortality in women, nor with specific causes of death. Restricted cubic splines analyses indicated that drinking up to 5 cups (or 625 ml)/day of tea was associated with significantly decreased mortality in men, with the lowest HR (0.72, 95% CI 0.57–0.91) observed in men drinking 2–3 cups/day, compared to nonconsumers. There was no significant interaction between coffee and tea intake. In substitution analyses, increasing tea intake (expressed as percentage of total coffee and tea intake) was significantly and nonlinearly related to lower overall, cancer and cardiovascular mortality in men, but in women higher tea percentages were positively associated with overall mortality (and most causes of death). This Dutch study suggests that for men, compared to exclusive coffee drinkers, those drinking 30–50% tea have the lowest mortality (HR 0.70); any nonzero percentage tea drinking seems better than only coffee. For women, those who drank exclusively coffee or drinking up to 40% tea had the lowest mortality, but those drinking higher percentages of tea were at increased mortality risk, up to a HR of 1.41 (1.01–1.99) for 80–100% tea drinkers).

The association between coffee and tea (and fluid) intake and mortality due to IHD or stroke was previously investigated in the NLCS [20]. Then, it was found that coffee was inversely associated with IHD mortality in women only (and positively in men), while tea intake was associated with lower IHD mortality in men only. For stroke mortality, no associations with coffee or tea were observed [20]. In this Dutch population, there is no difference between men and women in the way coffee and tea are prepared and consumed.

Several meta-analyses have been published on mortality and coffee and tea intake [4, 7, 21–25]. The most recent meta-analysis for coffee with 31 cohort studies [4] concluded that overall and CVD mortality were significantly reduced for consumption of up to 4 cups/day of coffee, with no further decrease in mortality with higher intakes. Nevertheless, there was significant heterogeneity in the estimates. When analyses were limited to non-smokers, cancer mortality was also decreased with increasing coffee intake. No relevant differences were noted according to gender, type of coffee, or geographical area. However, their restricted cubic splines analyses (Suppl Fig. 1 in Grosso et al. [4.]) also suggests that for cancer mortality, there was a clear inverse association with coffee in women, and no or a possibly positive association in men, similar to the current NLCS findings. In the NLCS we also found a significantly decreased overall mortality for intake up to 4–5 cups/day in women, but in men this was far less evident and not significant, and there was significant heterogeneity between the sexes. Recently, two major cohort studies also reported their findings on coffee and mortality [5, 6]. In EPIC, inverse associations with coffee intake were found with overall mortality in men and women, but coffee was positively associated with cancer mortality in women and not associated in men. For CVD mortality the association was much stronger inverse in women than in men [5]. In the MEC study, significantly inverse associations with increasing coffee intake were found with overall mortality in men and women in various nonwhite populations and in whites [6]. Inverse associations were also found with, a.o., deaths due to heart disease, cancer and respiratory disease, but no cause-specific results per sex were presented. Both studies did not additionally adjust for tea intake, nor reported on tests for nonlinearity in the associations with coffee. In another recent analysis [26] of three major cohorts, the Nurses’ Health Study (NHS) cohorts I and II, and Health Professionals Follow-up Study (HPFS), nonlinear associations between (total, caffeinated and decaffeinated) coffee intake and total mortality and most causes of death were seen, but not for cancer. Although the authors mentioned that results were not different for men and women, their sex-specific results for total mortality show that significantly inverse associations with coffee were only present for women in the NHS cohorts, but not for men in the HPFS. Unfortunately, no significance test was shown for the possible interaction with sex in that publication [26]. In the NIH-AARP Diet and Health Study [27] and the PLCO cohort [28], significant associations were seen for men and women. In the NLCS, associations with total mortality seem somewhat stronger inverse in women than in most other major cohorts and meta-analyses [4–6, 26, 27].

The most recent meta-analysis for tea with 18 cohort studies concluded that black tea consumption was significantly inversely associated with overall and cancer mortality, while green tea consumption was significantly inversely associated with overall and CVD mortality [7]. However, there was large and significant heterogeneity in the estimates for both types of tea. Both green and black tea showed significant nonlinear associations with overall mortality; for black tea this was a U-shaped relation with the lowest risk for those drinking 2–3 cups/day (of size 150 ml) [7]. This is comparable with the current NLCS findings among men. Due to the limited number of studies in the meta-analysis on black tea among men, differences according to gender could not be investigated very well. A recent analysis from the NHS II cohort [29] reported inverse associations between tea consumption (more than once per week versus nonconsumers) and total, cancer and CVD mortality, but no adjustment was made for coffee intake. It should be noted that the studied contrast in tea intake in NHS II (more than once per week versus nonconsumers) reflects the low tea intake levels in the NHS and is quite different from the levels studied in the NLCS (5+ cups/day versus nonconsumers) reflecting much higher intakes.

In a limited number of the previous studies, analyses for coffee were adjusted for tea intake, and vice versa. Surprisingly few studies have evaluated coffee and tea simultaneously in relation to mortality [30]. In the Northern Manhattan Study cohort (n = 2461, 36% men; 863 deaths), inverse associations for coffee and tea with total mortality were reported [30], and HRs for coffee were somewhat attenuated when other beverages were adjusted for, while associations for tea were strengthened. More so than coffee, tea was inversely associated with nonvascular mortality, particularly cancer death. They also found an inverse association of total intake of coffee and tea with mortality [30]. While no significant interaction was found between coffee and tea in the NLCS, the substitution analyses revealed that in men who only drink coffee had the highest mortality, and men drinking 30–50% of total coffee and tea intake as tea had the lowest mortality. Women who drank exclusively coffee or drank up to 40% tea had the lowest mortality, but mortality was increased at higher tea intake percentages. To the knowledge of the author, there is no other study that has investigated substitution effects of coffee versus tea on mortality. Before interpreting this (as causal), these differences in mortality risks associated with coffee and tea and per sex need confirmation first in other studies that can examine coffee and tea simultaneously.

Roasted coffee contains many bioactive compounds, the most important classes of which seem to be chlorogenic acids, caffeine, trigonelline and the diterpenes kahweol and cafestol [31]. In vitro experiments have shown antioxidant, anti-inflammatory, antihypertensive, hypoglycemic, and anticarcinogenic activity of these compounds [4, 31–35]. For example, caffeine might contribute to the antioxidant capacity of coffee. In addition, roasted coffee is an important source of nicotinic acid [36], but also of acrylamide which may exert adverse health effects [37]. For both coffee and tea, antioxidant mechanisms have been proposed as the most likely explanation of potential health benefit against chronic diseases, as well as anti-inflammatory mechanisms [4, 7]. The main polyphenols present in coffee are phenolic acids, particularly chlorogenic acids [4]. The main polyphenols present in tea are flavonoids, particularly catechins (in green tea) and flavanols, theaflavins and thearubigins in black tea [38–40]. It has been suggested that tea polyphenols could lower plasma cholesterol, inhibit reactive oxygen species, induce hypolipemia and decrease antifibrinolysis [7, 41, 42]. Cancer-protective effects of tea have been found in laboratory studies; the potential mechanisms responsible for this include modulation of phase II metabolism, increased antioxidant response, reduced inflammation through inhibition of NF-κB, inhibited growth factor (VEGF-1) signaling, and control of epigenetic modifications and modulation of immune system [7, 39, 43–45].

The observed differences for coffee and tea, according to gender, are potentially related to modulated endogenous sex hormone and sex hormone binding globulin (SHBG) levels. Only few studies have been conducted on coffee, tea and sex hormone and SHBG levels, and most were cross-sectional by design with varying results. Regarding cross-sectional studies, no significant association was found between coffee or caffeine intake and serum sex hormone and SHBG levels in 1241 middle-aged American men [46]. In a study among 52 healthy elderly Greek men, coffee intake was positively related to serum estradiol levels, but not to testosterone or SHBG levels [47]. In a study among 1563 Norwegian men, coffee was positively associated with SHBG and total testosterone levels [48]. A recent French study in 2377 women reported that (caffeinated) coffee, but not tea intake, was positively related with SHBG levels, notably in postmenopausal women [49], confirming earlier studies on coffee and SHBG levels in women. Low SHBG levels are considered as a consistent marker of type 2 diabetes risk, especially in women [50]. The estrogens estradiol and estrone are metabolized along three pathways, according to the initial hydroxylation at the 2-, 4- or 16-positions of the steroid ring [51]. In a study among 587 premenopausal US nurses focusing on coffee/tea intake and estrogen metabolism, coffee intake was associated with higher urinary 2-hydroxyestradiol and 2-hydroxyestrone levels, while tea intake was positively associated with 17-epiestriol levels (from the 16-pathway). Metabolites in the 2-pathway are hypothesized to have less genotoxic and estrogenic potential than metabolites form the 4- and 16-pathway, based on laboratory data [52]. The only randomized controlled trial on coffee and serum sex hormone levels found no consistent effect of caffeinated coffee on SHBG or sex hormones in men and women after 8 weeks, but the trial size was small (14 men and 28 women) [53]. Thus, associations between coffee and SHBG levels seem more consistent for women than men. Although several studies point towards possible differential associations of coffee and tea with sex hormone and SHBG levels in men and women, which might explain some of the observed mortality differences in the NLCS, the evidence is still inconsistent. More research is needed on this, especially from randomized controlled trials.

Strengths of the large-scale NLCS include the prospective design and high completeness of follow-up, which makes information and selection bias unlikely. The Dutch participants had both relatively high coffee and tea consumption levels with large variation between participants [3, 54], which enabled substitution analyses. Possible reverse causation due to changes in diet or lifestyle was minimized by excluding prevalent CVD or cancer cases [11]. Exclusion of early deaths from follow-up also did not change the results. Sensitivity analyses among stable coffee drinkers showed comparable associations, reducing the likelihood of reverse causation. The availability of detailed information on smoking habits also enabled better control for confounding. There was a large impact of multivariable adjustment on HRs, particularly with smoking; it is important to control for smoking as tightly as possible [15]. Associations with coffee and tea were also observed in the group of never smokers or those who stopped ≥ 10 years ago. Nevertheless, the possibility of residual confounding by smoking or confounding by unmeasured factors remains. Other limitations of the NLCS include the absence of updated information on coffee and tea intake during follow-up. Because there was no possibility to update dietary or other lifestyle data during follow-up, this may have resulted in some attenuated associations. However, analyses in other cohorts that have evaluated this with cumulatively updated measurements over time, no substantial changes in associations with coffee were seen [26]. The validation study of the food frequency questionnaire has shown that it performs relatively well [13], but measurement error may still have attenuated associations. Unfortunately, no specific validation study results were available relating to coffee and tea. Finally, only black tea could be evaluated, and no information on decaffeinated coffee was available in the NLCS.

In conclusion, coffee and tea intake were differently associated with total and cause-specific mortality in this cohort study, and associations varied between men and women. This was especially visible when substituting coffee with tea. Further research is needed to confirm or refute the mortality results in men and women obtained in the current study, and on possible differential effects of coffee and tea on sex hormone and SHBG levels.

## Electronic supplementary material

Below is the link to the electronic supplementary material.

Supplementary material 1 (PDF 420 kb)
