# Supplementary material for: Coffee or Tea? A prospective cohort study on the associations of coffee and tea intake with overall and cause-specific mortality in men versus women
Source: Eur J Epidemiol. 2018 Jan 27;33(2):183–200. doi: 10.1007/s10654-018-0359-y (PMC5871637; doi:10.1007/s10654-018-0359-y)
Supplement: Supplementary file 1 — Supplementary material 1 (PDF 420 kb) [file 10654_2018_359_MOESM1_ESM.pdf]

SUPPLEMENTARY MATERIALS

European Journal of Epidemiology

Article Title:

Coffee or Tea?

A prospective cohort study on the associations of coffee and tea intake with overall and cause-specific mortality in men versus women

Piet A. van den Brandt[1,2]

[1] Maastricht University Medical Centre, GROW- School for Oncology and Developmental Biology, Department of Epidemiology, Maastricht, the Netherlands

[2] Maastricht University Medical Centre, CAPHRI- School for Public Health and Primary Care, Department of Epidemiology, Maastricht, the Netherlands

Address for correspondence:

Piet A. van den Brandt, PhD;

Maastricht University Medical Centre; Department of Epidemiology; PO Box 616; 6200 MD Maastricht; The Netherlands; Tel.: +31 (0)43 3882361; E-mail:

PA.vandenBrandt@maastrichtuniversity.nl

Supplementary Table 1. Overall mortality by categories of intake of coffee and tea in men and women respectively, in multivariable-adjusted[a] interaction analyses.

	Tea (cups/day) (median)			*P*
Coffee (cups/day)	0-<2 cups/d	2-<4 cups/d	4+ cups/d	heterogeneity
Men				
5+ cups/day				0.908
Deaths / Person-yrs	1159 / 2502	954 / 2483	587 / 1466	
Multivariable-adjusted HR	1 (Ref)	0.82	0.77	
(95% CI)		(0.65 - 1.03)	(0.59 - 1.01)	
2-<5 cups/day				
Deaths / Person-yrs	625 / 1464	1056 / 2619	954 / 2382	
Multivariable-adjusted HR	0.90	0.79	0.81	
(95% CI)	(0.69 - 1.19)	(0.62 - 1.00)	(0.63 - 1.04)	
0-<2 cups/day				
Deaths / Person-yrs	61 / 163	76 / 259	164 / 451	
Multivariable-adjusted HR	0.84	0.74	0.86	
(95% CI)	(0.43 - 1.63)	(0.45 - 1.22)	(0.57 - 1.30)	
Women				
5+ cups/day				0.422
Deaths / Person-yrs	344 / 1588	329 / 2127	268 / 1455	
Multivariable-adjusted HR	1 (Ref)	0.91	1.04	
(95% CI)		(0.67 - 1.24)	(0.73 - 1.47)	
2-<5 cups/day				
Deaths / Person-yrs	289 / 1676	689 / 3735	818 / 4399	
Multivariable-adjusted HR	0.90	1.05	1.07	
(95% CI)	(0.64 - 1.25)	(0.79 - 1.39)	(0.80 - 1.44)	
0-<2 cups/day				
Deaths / Person-yrs	34 / 115	77 / 245	181 / 767	
Multivariable-adjusted HR	2.11	1.92	1.40	
(95% CI)	(0.99 - 4.53)	(1.06 - 3.45)	(0.93 - 2.09)	

[a] Multivariable analyses were adjusted for: age at baseline (continuous, in years), cigarette smoking status (coded as never, former, current smoker), number of cigarettes smoked per day, and years of smoking (both continuous, centered)), history of physician-diagnosed hypertension (no, yes) and diabetes (no, yes), body height (continuous, m), BMI (<18.5, 18.5-<25, 25-<30, ≥30 kg/m2), non-occupational physical activity (<30, 30-60, 61-90, ≥90 min/day), highest level of education (primary school or lower vocational, secondary or medium vocational, and higher vocational or university), intake of alcohol (0, 0.1-<5, 5-<15, 15-<30, 30+ g/day), nuts (0, 0.1-<5, 5-<10, 10+ g/day), vegetables and fruit (both continuous, g/day), energy (continuous, kcal/day), use of nutritional supplements (no, yes), and, in women, postmenopausal HRT (never, ever).

Supplementary Figure S1. Flow diagram of the number of subcohort members and deaths on which analyses are based, Netherlands Cohort Study.

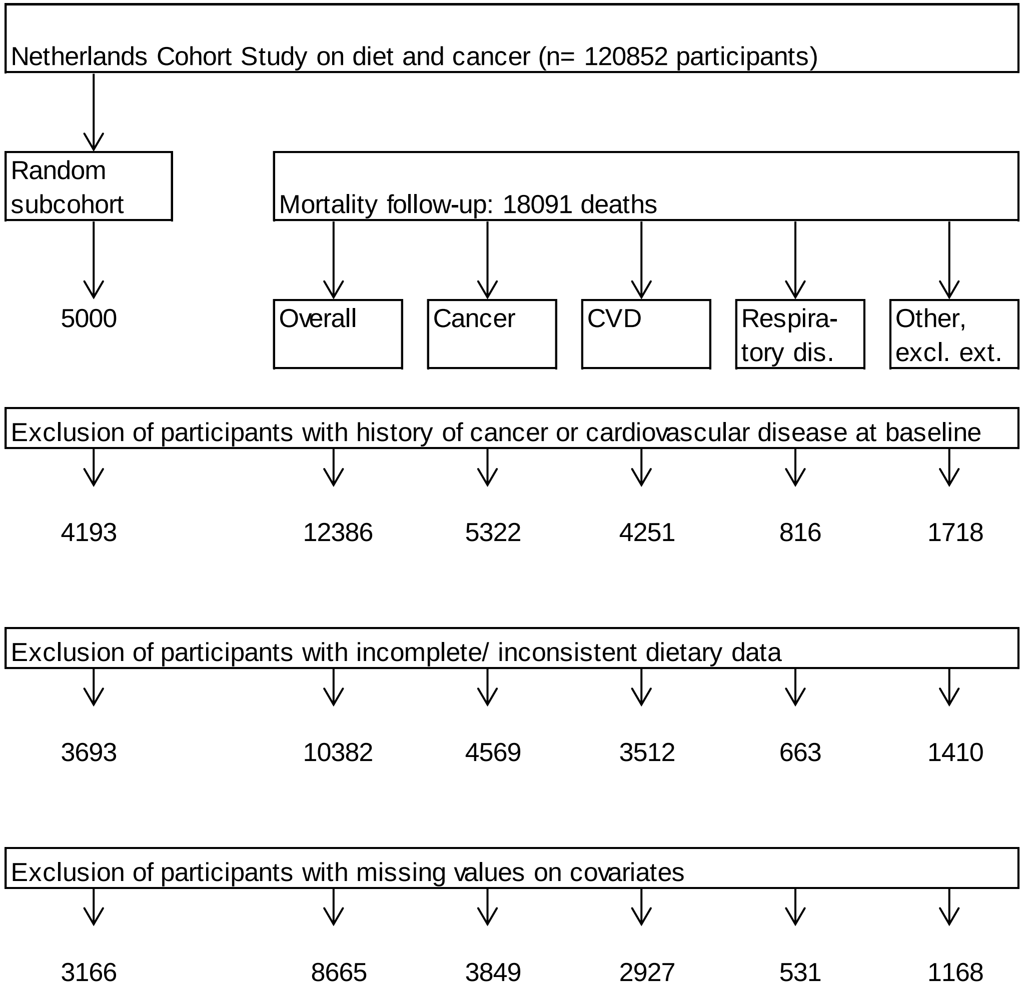

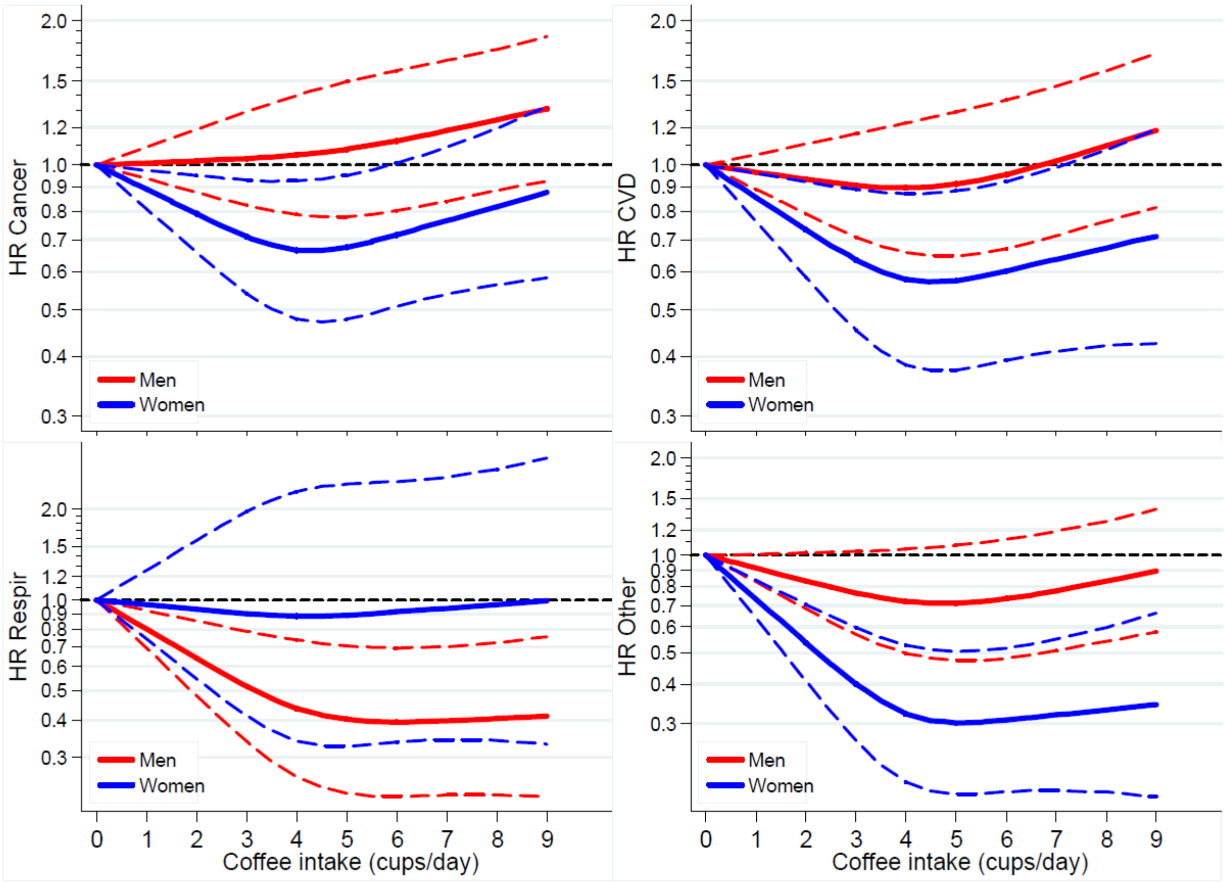

Supplementary Figure S2. Spline regression curves for the association between coffee intake and cause-specific mortality. Red lines: men. Blue lines: women.
Multivariate HRs are calculated by restricted cubic spline regression (using 3 knots at 10th, 50th, and 90th percentiles) adjusting for: age at baseline (continuous, in years), cigarette smoking status (coded as never, former, current smoker), number of cigarettes smoked per day, and years of smoking (both continuous, centered), history of physician-diagnosed hypertension (no, yes) and diabetes (no, yes), body height (continuous, m), BMI (<18.5, 18.5-<25, 25-<30, ≥30 kg/m2), non-occupational physical activity (<30, 30-60, 61-90, ≥90 min/day), highest level of education (primary school or lower vocational, secondary or medium vocational, and higher vocational or university), intake of alcohol (0, 0.1-<5, 5-<15, 15-<30, 30+ g/day), nuts (0, 0.1-<5, 5-<10, 10+ g/day), vegetables and fruit (both continuous, g/day), tea (continuous, cups/day), energy (continuous, kcal/day), use of nutritional supplements (no, yes), and, in women, postmenopausal HRT (never, ever).

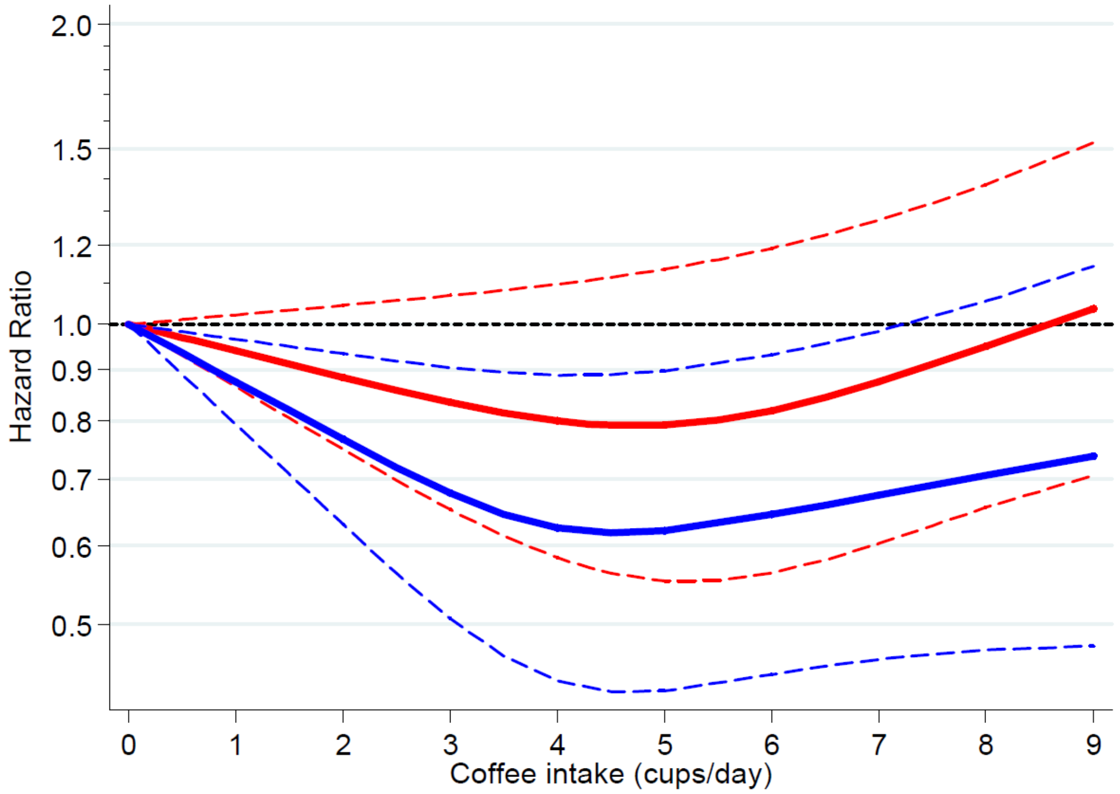

Supplementary Fig S3. Nonparametric regression curves for the association between coffee intake and total mortality, among stable coffee drinkers. Red lines: men. Blue lines: women. Solid lines represents point estimates and dashed lines represent 95% confidence intervals. Multivariate HRs are calculated by restricted cubic spline regression (using 3 knots at 10th, 50th, and 90th percentiles) adjusting for: age at baseline (continuous, in years), cigarette smoking status (coded as never, former, current smoker), number of cigarettes smoked per day, and years of smoking (both continuous, centered), history of physician-diagnosed hypertension (no, yes) and diabetes (no, yes), body height (continuous, m), BMI (<18.5, 18.5-<25, 25-<30, ≥30 kg/m2), non-occupational physical activity (<30, 30-60, 61-90, ≥90 min/day), highest level of education (primary school or lower vocational, secondary or medium vocational, and higher vocational or university), intake of alcohol (0, 0.1-<5, 5-<15, 15-<30, 30+ g/day), nuts (0, 0.1-<5, 5-<10, 10+ g/day), vegetables and fruit (both continuous, g/day), tea (continuous, cups/day), energy (continuous, kcal/day), use of nutritional supplements (no, yes), and, in women, postmenopausal HRT (never, ever).

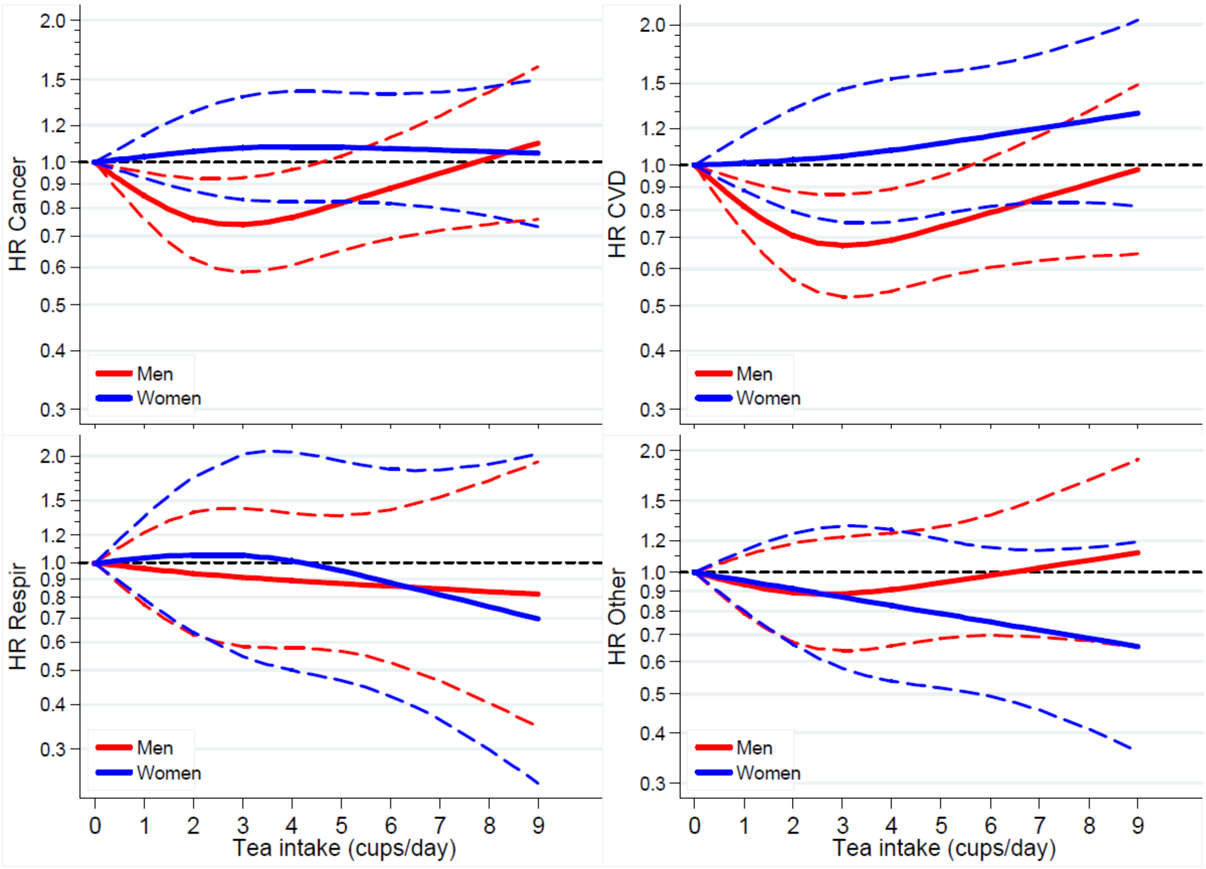

Supplementary Fig S4. Spline regression curves for the association between tea intake and cause-specific mortality. Red lines: men. Blue lines: women.
Multivariate HRs are calculated by restricted cubic spline regression (using 3 knots at 10th, 50th, and 90th percentiles) adjusting for: age at baseline (continuous, in years), cigarette smoking status (coded as never, former, current smoker), number of cigarettes smoked per day, and years of smoking (both continuous, centered), history of physician-diagnosed hypertension (no, yes) and diabetes (no, yes), body height (continuous, m), BMI (<18.5, 18.5-<25, 25-<30, ≥30 kg/m2), non-occupational physical activity (<30, 30-60, 61-90, ≥90 min/day), highest level of education (primary school or lower vocational, secondary or medium vocational, and higher vocational or university), intake of alcohol (0, 0.1-<5, 5-<15, 15-<30, 30+ g/day), nuts (0, 0.1-<5, 5-<10, 10+ g/day), vegetables and fruit (both continuous, g/day), coffee (continuous, cups/day), energy (continuous, kcal/day), use of nutritional supplements (no, yes), and, in women, postmenopausal HRT (never, ever).

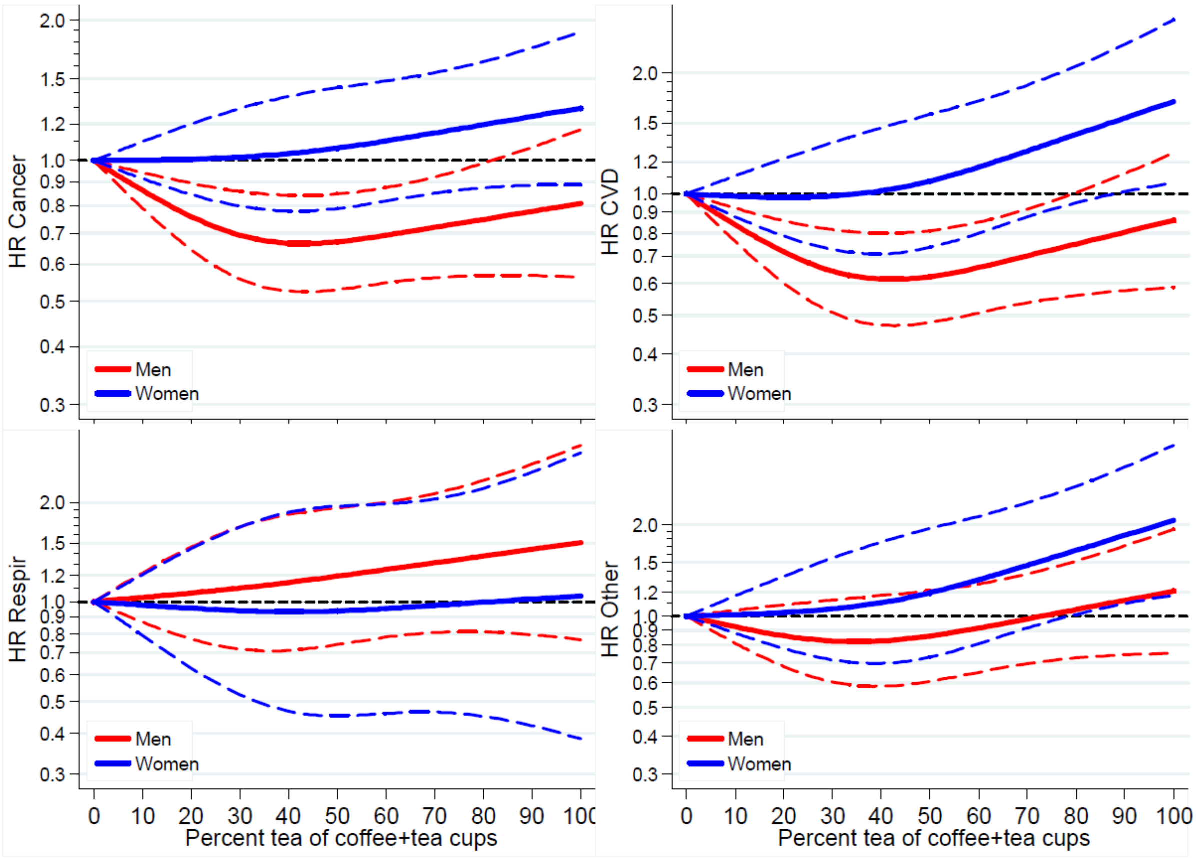

Supplementary Fig S5. Spline regression curves for the association between percentage tea of total coffee and tea and cause-specific mortality in substitution analyses. Red lines: men. Blue lines: women.

Multivariate HRs are calculated by restricted cubic spline regression (using 3 knots at 10th, 50th, and 90th percentiles) adjusting for: age at baseline (continuous, in years), cigarette smoking status (coded as never, former, current smoker), number of cigarettes smoked per day, and years of smoking (both continuous, centered), history of physician-diagnosed hypertension (no, yes) and diabetes (no, yes), body height (continuous, m), BMI (<18.5, 18.5-<25, 25-<30, ≥30 kg/m2), non-occupational physical activity (<30, 30-60, 61-90, ≥90 min/day), highest level of education (primary school or lower vocational, secondary or medium vocational, and higher vocational or university), intake of alcohol (0, 0.1-<5, 5-<15, 15-<30, 30+ g/day), nuts (0, 0.1-<5, 5-<10, 10+ g/day), vegetables and fruit (both continuous, g/day), coffee+tea (continuous, cups/day), energy (continuous, kcal/day), use of nutritional supplements (no, yes), and, in women, postmenopausal HRT (never, ever).

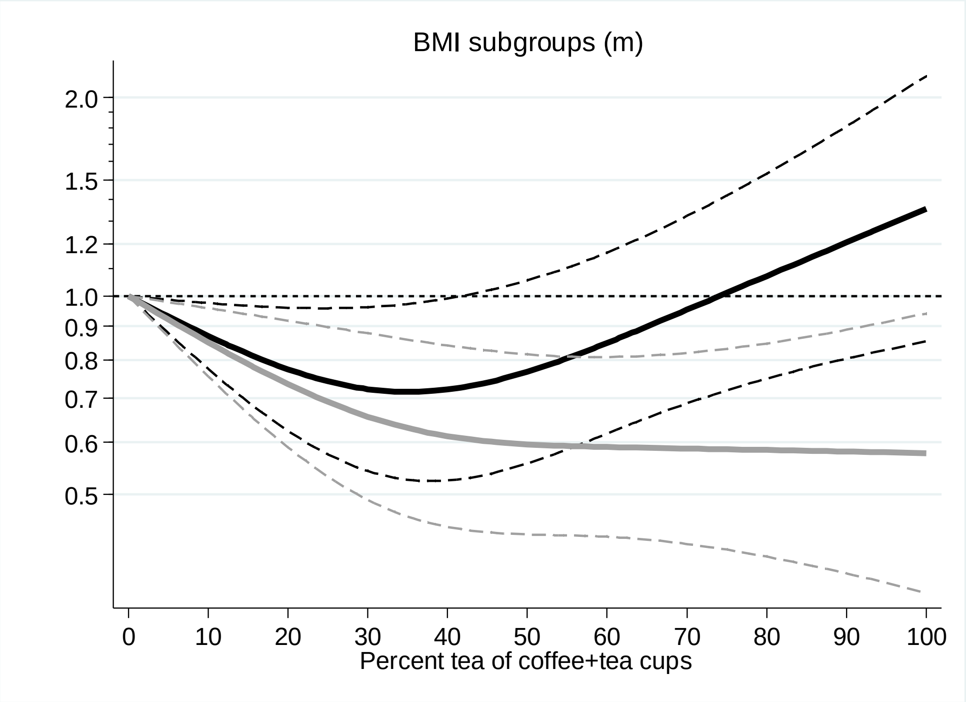

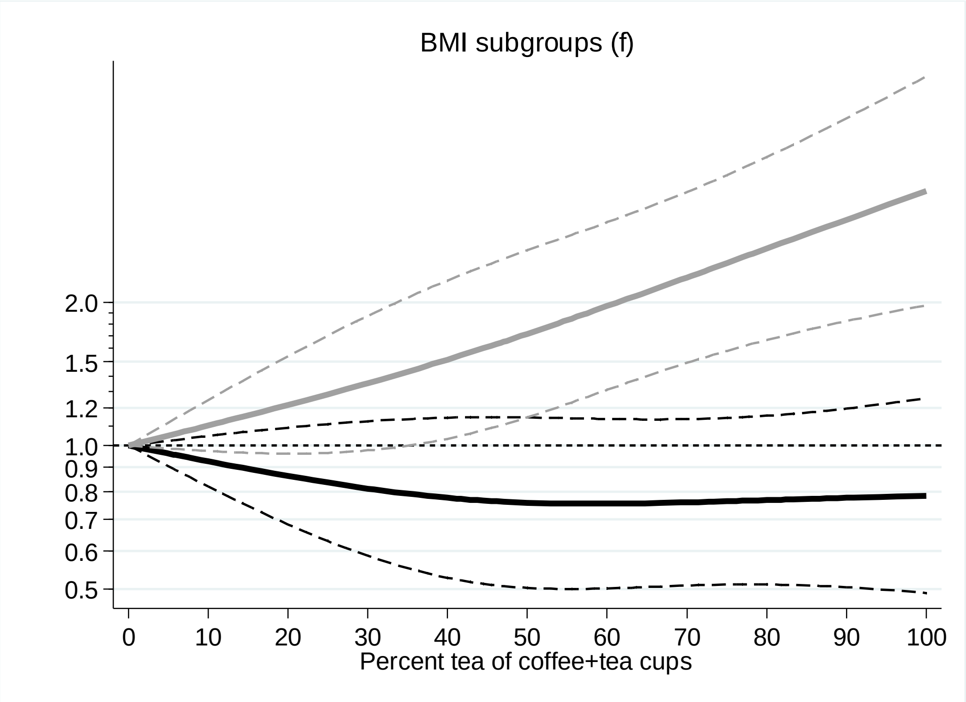

Supplementary Fig S6. Nonparametric regression curves for the association between percentage tea of total coffee and tea and total mortality in subgroups of BMI, in males and females, respectively. Black lines: BMI category 18.5-25 kg/m^2. Grey lines: BMI category 25+ kg/m^2. Multivariate HRs are calculated by restricted cubic spline regression (using 3 knots at 10th, 50th, and 90th percentiles) adjusting for: age at baseline (continuous, in years), cigarette smoking status (coded as never, former, current smoker), number of cigarettes smoked per day, and years of smoking (both continuous, centered), history of physician-diagnosed hypertension (no, yes) and diabetes (no, yes), body height (continuous, m), non-occupational physical activity (<30, 30-60, 61-90, ≥90 min/day), highest level of education (primary school or lower vocational, secondary or medium vocational, and higher vocational or university), intake of alcohol (0, 0.1-<5, 5-<15, 15-<30, 30+ g/day), nuts (0, 0.1-<5, 5-<10, 10+ g/day), vegetables and fruit (both continuous, g/day), coffee+tea (continuous, cups/day), energy (continuous, kcal/day), use of nutritional supplements (no, yes), and, in women, postmenopausal HRT (never, ever).

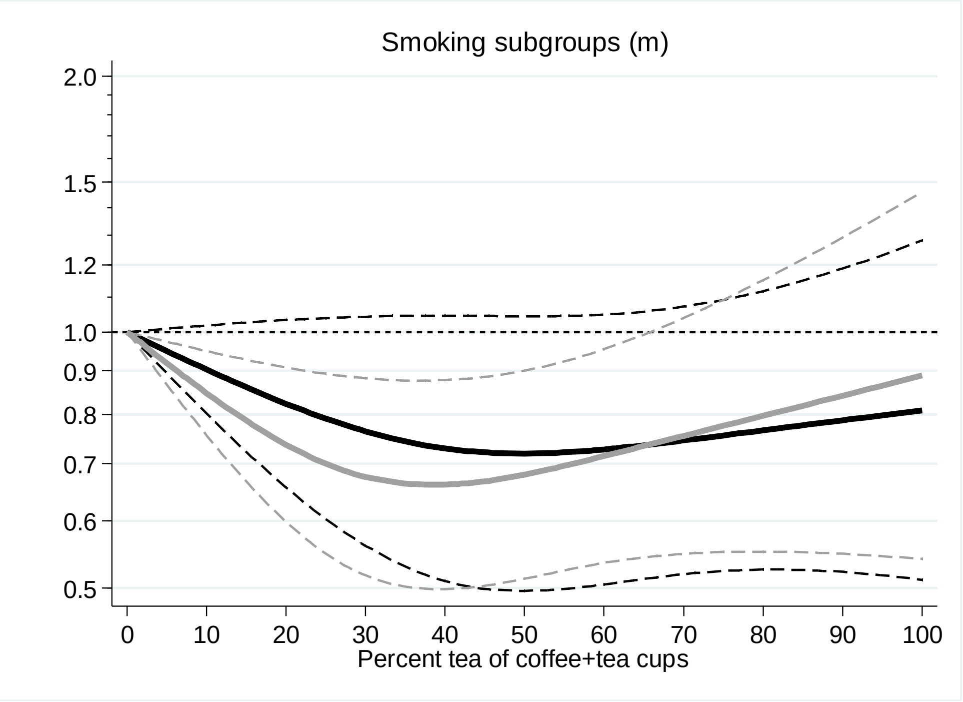

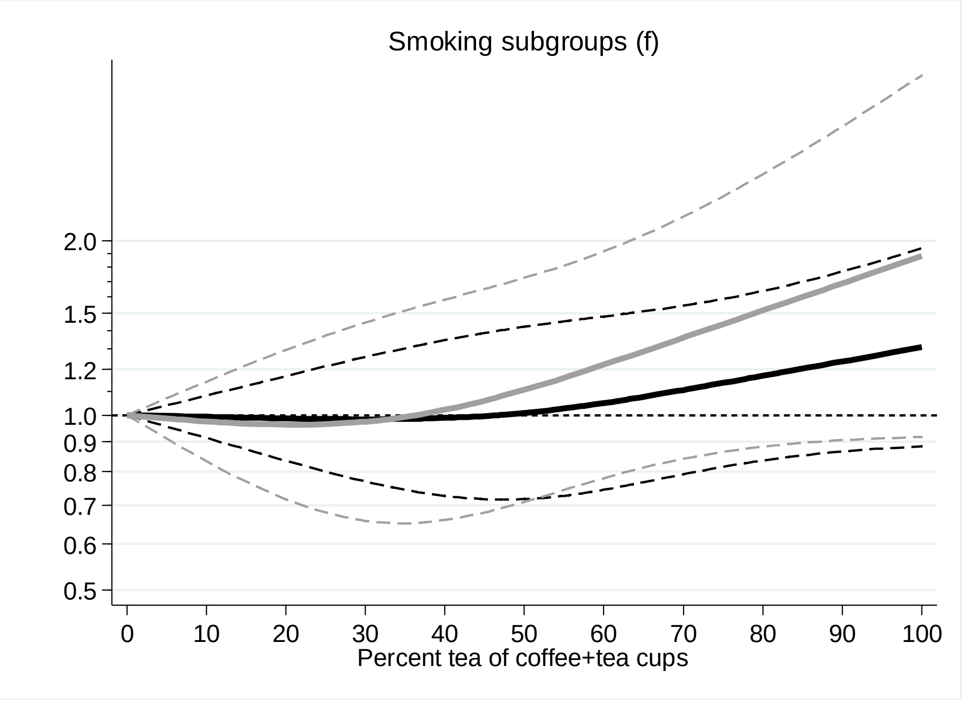

Supplementary Fig S7. Nonparametric regression curves for the association between percentage tea of total coffee and tea and total mortality in subgroups of smoking, in males and females, respectively. Black lines: Never smokers or those who stopped ≥10 y ago. Grey lines: Current smokers or stopped <10 y ago.

Multivariate HRs are calculated by restricted cubic spline regression (using 3 knots at 10th, 50th, and 90th percentiles) adjusting for: age at baseline (continuous, in years), number of cigarettes smoked per day and years of smoking (both continuous, centered), history of physician-diagnosed hypertension (no, yes) and diabetes (no, yes), body height (continuous, m), BMI (<18.5, 18.5-<25, 25-<30, ≥30 kg/m2), non-occupational physical activity (<30, 30-60, 61-90, ≥90 min/day), highest level of education (primary school or lower vocational, secondary or medium vocational, and higher vocational or university), intake of alcohol (0, 0.1-<5, 5-<15, 15-<30, 30+ g/day), nuts (0, 0.1-<5, 5-<10, 10+ g/day), vegetables and fruit (both continuous, g/day), coffee+tea (continuous, cups/day), energy (continuous, kcal/day), use of nutritional supplements (no, yes), and, in women, postmenopausal HRT (never, ever).
